# Intermittent Fasting and High-Intensity Exercise Elicit Sexual-Dimorphic and Tissue-Specific Adaptations in Diet-Induced Obese Mice

**DOI:** 10.3390/nu12061764

**Published:** 2020-06-12

**Authors:** Robin A. Wilson, Christos G. Stathis, Alan Hayes, Matthew B. Cooke

**Affiliations:** 1 Institute for Health and Sport, Victoria University, Footscray, VIC 3011, Australia; robin.wilson1@live.vu.edu.au (R.A.W.); Christos.Stathis@vu.edu.au (C.G.S.); Alan.Hayes@vu.edu.au (A.H.); 2 Australian Institute for Musculoskeletal Science (AIMSS), Victoria University, St Albans, VIC 3021, Australia; 3 Department of Medicine-Western Health, Melbourne Medical School, The University of Melbourne, St Albans, VIC 3021, Australia; 4 Department of Health and Medical Sciences, Swinburne University of Technology, Hawthorn, VIC 3122, Australia

**Keywords:** microRNA, genes, adipose, hypothalamus, muscle

## Abstract

The molecular adaptations that underpin body composition changes and health benefits of intermittent fasting (IF) and high-intensity interval training (HIIT) are unclear. The present study investigated these adaptations within the hypothalamus, white adipose and skeletal muscle tissue following 12 weeks of IF and/or HIIT in diet-induced obese mice. Mice (C57BL/6, 8-week-old, males/females) were fed high-fat (59%) and sugar (30%) water (HF/S) for 12 weeks followed by an additional 12 weeks of HF/S plus either IF, HIIT, combination (IF+HIIT) or HF/S only control (CON). Tissues were harvested at 12 and 24 weeks and analysed for various molecular markers. Hypothalamic *NPY* expression was significantly lower following IF+HIIT compared to CON in females. In adipose tissue, leptin expression was significantly lower following IF and IF+HIIT compared to CON in males and females. Males demonstrated increased markers of fat oxidation (*HADH*, *FABP4*) following IF+HIIT, whereas females demonstrated reduced markers of adipocyte differentiation/storage (*CIDEC* and *FOXO1*) following IF and/or IF+HIIT. In muscle, *SIRT1*, *UCP3, PGC1α*, and *AS160* expression was significantly lower following IF compared to CON in males and/or females. This investigation suggests that males and females undertaking IF and HIIT may prevent weight gain via different mechanisms within the same tissue.

## 1. Introduction

In the past century, there has been a noticeable transition in the nutritional habits of Western society towards increased consumption of animal-derived foods, saturated fats and refined sugars, with concomitant reduction of lean meats, high-fibre foods, vegetables and fruits [1,2]. These altered eating habits combined with reduced physical activity and energy expenditure appear to be a major contributor to the current obesogenic lifestyle [3]. With obesity rates on the rise, these lifestyle habits are also negatively impacting the function of many tissues including the brain, adipose tissue, liver, and skeletal muscle, leading to an increase in disorders such as metabolic syndrome, diabetes and cardiovascular disease. The hypothalamus, which is home to the arcuate nucleus and hypothalamic Neuropeptide Y/Agouti-Related Peptide (NPY/AgRP) and pro-opiomelanocortin/cocaine- and amphetamine-regulated transcript (POMC/CART) neurons, is one of the first tissues impacted by the obesogenic lifestyle [4]. Activation of inflammatory mediators occurs rapidly upon consumption of high-fat diets, even prior to significant weight gain or peripheral inflammation [5,6]. While this acute inflammatory response can recede after a few days, it typically returns with prolonged high-fat feeding and obesity status. Chronic inflammation can potentially alter neurocircuit functions and uncouple regulation of caloric intake and energy expenditure, promoting overeating and further weight gain [4].

Adipose tissue is also impacted by prolonged overnutrition and sedentary lifestyle. Pathologic remodelling and expansion of adipocytes, chronic inflammation, and fibrosis can cause tissue dysfunction and failure to store excess lipids. Consequently, excess lipids are deposited into non-adipose tissues, with ectopic storage of triglycerides in skeletal muscle linked to insulin resistance [7]. This sequence of events, starting with neuropathological alterations of the hypothalamic area, disturbed energy homeostasis and peripheral tissue dysfunction, is integral to the development of obesity and associated co-morbidities. Further, interplay between genetic predisposition and environmental factors, including complex regulatory systems involving epigenetic factors may also be perpetuating these potentially reversible processes. Therefore, interventions that can reduce weight and associated inflammation and reverse the modulation of key genetic mediators within a large number and variety of integrated organs may be critical in combating the obesity epidemic.

Popular lifestyle interventions such as intermittent fasting (IF) and high-intensity interval training (HIIT) are effective in reducing body weight and fat over a short to medium timeframe in humans [8,9]. We recently showed that both strategies, independently and combined, can attenuate the weight- and/or disease-promoting effects typically observed in a mice model of diet-induced obesity while consuming a high-fat and sugar diet (HF/S) [10]. Though the anthropometric and metabolic improvements following these lifestyle interventions are evident, understanding of the molecular pathways, including epigenetic regulators, that are responsive to diet and/or exercise and contributing to these adaptations is still lacking. Therefore, to help understand such mechanisms, we are subsequently investigating the molecular pathways that regulate appetite, energy homeostasis and inflammation within the hypothalamus; and structure, metabolic function and inflammation within white adipose tissue (WAT) and skeletal muscle. Further, we aim to identify which molecular changes correlate with our previously published anthropometric and metabolic health measurements [10]. We hypothesise that HIIT and IF will attenuate the expression of key molecular pathways that drive weight gain, glucose tolerance and/or dyslipidaemia as a result of reduced inflammation and/or change in energy status of the tissue. Additionally, the combination of diet and exercise will provide a synergistic effect and demonstrate greater changes compared to either intervention alone.

## 2. Materials and Methods

### 2.1. Previous Published Materials and Methods

The original Materials and Methods of this study have been published elsewhere [10]. This includes full details regarding the housing and feeding of animals, diet and exercise protocols used in accordance with ethical approval obtained from the Victoria University Animal Ethics Committee (AEC15/007). In the current paper, we will present a brief overview of the animals and supplement groups used. We have provided details about the processing of tissues and subsequent analysis, not published elsewhere.

### 2.2. Animals

Eight-week-old male and female mice (C57BL/6, males = 39 and females = 49) were fed a high-fat (HF) (59% energy from fat, SF03-002, Speciality Feeds, Glen Forrest, WA, Australia) and sugar (S) water (30% sugar w/v) diet for a period of 12 weeks to induce obesity. One group (males = 8, females = 10) was culled at 12 weeks to serve as the ‘baseline’ comparison group (OBC) for the 24 week CON group and to determine the time effects of a further 12 week of the HF/S diet. All other mice continued consuming the HF/S diet for another 12 weeks, but were randomly allocated (block randomisation) into 1 of 4 groups: no intervention, HF/S only (CON; males = 7, females = 9); intermittent fasting diet (IF; males = 8, females = 10); high-intensity interval training (HIIT; males = 8, females = 10); and a combination of the diet and exercise (IF + HIIT; males = 8, females = 10). At the end of the additional 12 week diet and intervention period, mice (now 32 weeks old) were anaesthetised with intraperitoneal injection of pentobarbitone (60 mg/kg). The left extensor digitorum longus (EDL) muscle was isolated, removed and snap frozen in liquid nitrogen. Given the high-intensity nature of the exercise training, the fast twitch EDL muscle was chosen as most appropriate for investigation. Mice were then killed by cardiac puncture and the abdominal cavity was opened to collect epididymal (males) and periovarian (females) WAT, which was snap frozen in liquid nitrogen. Whole brain was isolated and snap frozen in liquid nitrogen. Just before RNA extraction, brain was placed in RNAlater solution on ice and hypothalamus was isolated quickly using scalpel. All samples were stored at −80 °C until further analysis.

### 2.3. mRNA and miRNA Expression

Total RNA was extracted from WAT and EDL muscle using an miRNeasy mini kit (Qiagen); and from hypothalamus using a RNeasy lipid tissue mini kit (Qiagen). The RNA content was measured using a nano-drop spectrophotometer (DeNovix DS-11, DeNovix, USA) and RNA quality was assessed using the A_260_/A_280_ ratio. The average ratio for all RNA samples was 1.99. RNA integrity was determined using agarose gel electrophoresis with a SYBR™ Green II RNA gel stain (Invitrogen). The 28s and 18s rRNA band ratio between 1.3 and 2 was taken as measure for good RNA quality. RNA was reverse transcribed to generate cDNA using a RT^2^ first strand kit (Qiagen) from mRNA and a miScript II RT kit (Qiagen) from miRNA and subjected to RT-PCR for gene and microRNA (miRNA/miR) expression using a RT^2^ SYBR green qPCR mastermix and a miScript SYBR green PCR kit, respectively. Specific microRNAs were selected depending on their relationship with the genes of interest investigated in the present study, based on either previous studies reporting those gene–miRNA relationships or based on the miRWALK database [11]. Beta-2 microglobulin (*B2M*), peptidylprolyl isomerase A (*PPIA*) and RNU6 were used to normalize mRNA expression in WAT, in muscle, and miRNA expression in both adipose and muscle, respectively. The average beta-actin (β actin), *PPIA* and glyceraldehyde-3-phosphate dehydrogenase (GAPDH) expression was used to normalize hypothalamus mRNA expression. mRNA/miRNA expression was calculated using the ΔΔCt method. The primers for genes and miRNAs were purchased from and designed by QIAGEN (Chadstone, Victoria, Australia). The list of genes and their purported function, and miRNAs analysed is in Supplementary Tables S1 and S2, respectively.

### 2.4. Statistical Analysis

Data is presented as the mean ± standard deviation (SD). The mRNA and miRNA expression levels of the CON group are presented relative to the OBC group and was compared using an unpaired Students T-test. The mRNA and miRNA expression in interventions groups (HIIT, IF, and IF+HIIT) are presented relative to the CON group and differences were analysed using a one-way ANOVA with Tukey’s post-hoc test for subsequent analysis. Anthropometric and health parameters presented in the Wilson et al. study [10] were correlated to mRNA and miRNA expression data using Pearson correlation analysis. The correlation tables are given in Supplementary Tables (Tables S3–S7). All the statistical analysis was performed using GraphPad Prism 8.4.1. An alpha level of 0.05 was adopted throughout to reduce the chance for Type I statistical error.

## 3. Results

### 3.1. Previously Published Results

**Figure 1:**
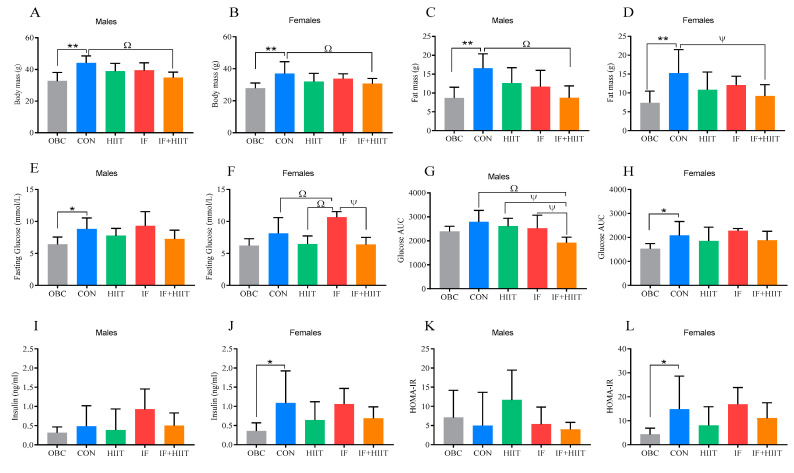
Body weight (g) (**A**,**B**), fat mass (g) (**C**,**D**), fasting glucose (mmol/L) (**E**,**F**), glucose area under the curve (**G**,**H**), plasma insulin (ng/ml) (**I**,**J**) and homeostatic model assessment of insulin resistance (HOMA-IR) (**K**,**L**) in males and females, respectively. Bars represent the mean, and error bars represent the standard deviation. * and ** represent a significant difference between the OBC and the CON group at *p* < 0.05 and *p* < 0.01, respectively. Ψ and Ω represent a significant difference between the CON and IF, HIIT and IF+HIIT groups at *p* < 0.05 and *p* < 0.01, respectively.

Body composition, strength and blood variables of this study have been published elsewhere [10]. We have performed Pearson correlation analysis on selected molecular targets that demonstrated significant change at 24 weeks following diet and/or exercise intervention with previously published body composition and blood variable data [10]. Selected body composition and blood values are presented again (Figure 1 and Figure S1), but in a different way to the previously published format, for the reader’s reference when interpreting the correlation analysis. Body weight and fat mass was significantly higher in the CON group compared to the OBC group in both males and females (*p* < 0.01). At the end of intervention period, body weight in the IF+HIIT group was significantly lower than the CON group (*p* < 0.01) in both males and females. Fat mass was also significantly lower in the IF+HIIT group compared to CON in males (*p* < 0.01) and females (*p* < 0.05). In males, fasting glucose in the OBC group was significantly lower than in the CON group (*p* < 0.05). In females, fasting glucose in the CON and HIIT group (*p* < 0.01) and the IF+HIIT (*p* < 0.05) group was significantly lower than in the IF group. Glucose area under the curve (AUC) was lower in the IF+HIIT group compared to the CON (*p* < 0.01), HIIT and IF groups (*p* < 0.05) in males. In females, glucose AUC, fasting insulin and Homeostatic model assessment of insulin resistance (HOMA-IR) in the OBC group were significantly lower than in the CON group (*p* < 0.05). 

### 3.2. The Effect of the HF/S Diet on Hypothalamic mRNA Expression

**Figure 2:**
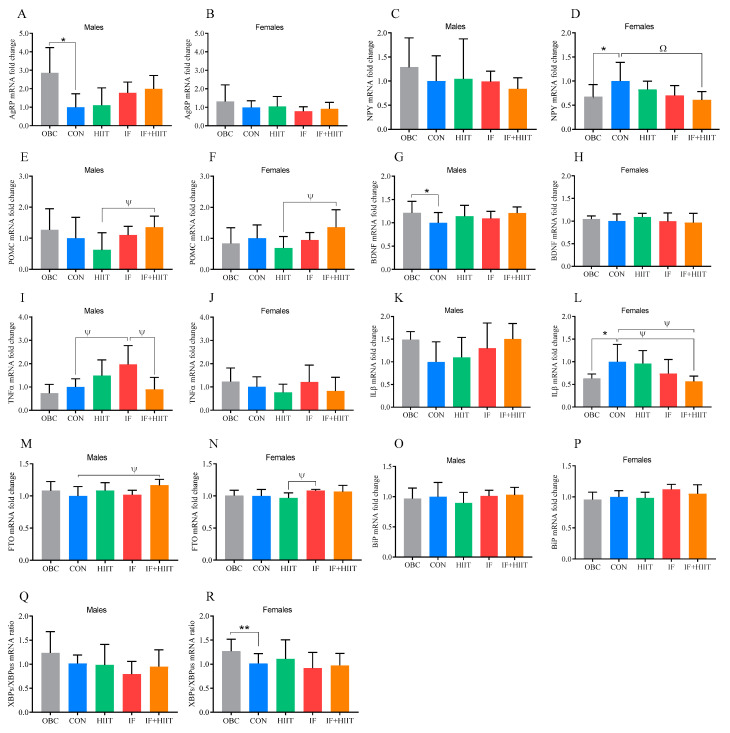
Hypothalamus gene expression of the OBC, IF, HIIT and IF+HIIT groups relative to CON. Relative gene expression of *AgRP* in (**A**) males and (**B**) females, *NPY* in (**C**) males and (**D**) females, *POMC* in (**E**) males and (**F**) females, *BDNF* in (**G**) male and (**H**) females, TNFα in (**I**) males and (**J**) females, *IL1β* in (**K**) males and (**L**) females, *FTO* in (**M**) males and (**N**) females, *BiP* in (**O**) males and (**P**) females and *XBPs*/*XBPus* ratio in (**Q**) males and (**R**) females. Gene expression is normalized to the average *ACTB*, *GAPDH* and *PPIA* gene expression. Bars represent the mean, and error bars represent the standard deviation. * and ** represent a significant difference between the OBC and the CON group at *p* < 0.05 and *p* < 0.01, respectively. Ψ and Ω represent a significant difference between the CON and IF, HIIT and IF+HIIT groups at *p* < 0.05 and *p* < 0.01, respectively.

Following an additional 12 weeks of the HF/S diet, AgRP and BDNF expression was significantly lower in the CON group compared to the OBC group in male mice (*p* < 0.05, Figure 2A,G). In females, NPY and IL1-β expression was significantly higher in the CON group compared to the OBC group (*p* < 0.05, Figure 2D,L), whereas XBPus/XBPs was significantly lower compared to OBC (*p* < 0.01, Figure 2R).

### 3.3. The Effect of IF and/or HIIT on Hypothalamic mRNA Expression

At the end of the 12 week intervention, *NPY* expression was significantly lower in the IF+HIIT group compared to CON in females only (*p* < 0.01, Figure 2D). NPY expression was also positively correlated with body weight (r = 0.402, *p* < 0.05) and body fat (r = 0.422, *p* < 0.01). Conversely, *POMC* expression was higher in the IF+HIIT group, but only statistically significant compared to the HIIT group in both males (*p* < 0.05, Figure 2E) and females (*p* < 0.05, Figure 2F). FTO expression was significantly higher in the IF+HIIT group compared to the CON group in males (*p* < 0.05, Figure 2M), whereas females demonstrated significant differences in *FTO* expression between the IF group and the HIIT group (*p* < 0.05, Figure 2N). *TNFα* was significantly higher in the IF group compared to the CON and IF+HIIT groups in males (*p* < 0.05 Figure 2I). TNFα expression was positively correlated to HOMA-IR level in males (r = 0.501, *p* < 0.01). *IL1β* expression was significantly lower in the IF+HIIT group compared to the CON and HIIT groups in females (*p* < 0.05, Figure 2L).

### 3.4. The Effect of the HF/S Diet on White Adipose Tissue mRNA Expression

**Figure 3:**
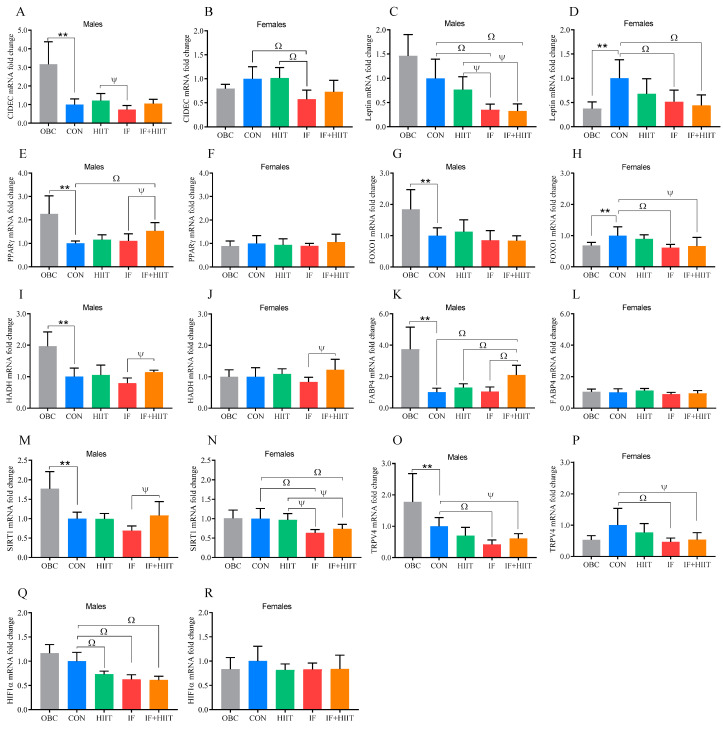
White adipose tissue gene expression of the OBC, IF, HIIT and IF+HIIT groups relative to CON. Relative gene expression of *CIDEC* in (**A**) males and (**B**) females, leptin in (**C**) males and (**D**) females, *PPARγ* in (**E**) males and (**F**) females, *FOXO1* in (**G**) male and (**H**) females, *HADH* in (**I**) males and (**J**) females, *FABP4* in (**K**) males and (**L**) females, *SIRT1* in (**M**) males and (**N**) females, *TRPV4* in (**O**) males and (**P**) females and *HIF1α* in (**Q**) males and (**R**) females. Gene expression is normalized to *B2M* gene expression. Bars represent the mean, and error bars represent the standard deviation. ** represents a significant difference between the OBC and the CON group at *p* < 0.01. Ψ and Ω represent a significant difference between the CON and IF, HIIT and IF+HIIT groups at *p* < 0.05 and *p* < 0.01, respectively.

Following an additional 12 weeks of the HF/S diet, *CIDEC*, *PPARγ FOXO1*, *HADH*, *FABP4*, *SIRT1*, and *TRPV4* expression was significantly lower in the CON group compared to the OBC group in male mice (*p* < 0.01, Figure 3A,E,G,I,K,M,O). In female mice, leptin and *FOXO1* expression was significantly higher in the CON group compared to the OBC group (*p* < 0.01, Figure 3D,H).

### 3.5. The Effects of IF and/or HIIT on White Adipose Tissue mRNA Expression

At the end of the 12 week intervention, *CIDEC* expression was significantly lower in the IF group compared to the CON group in females (*p* < 0.01) and in the HIIT group in both males (*p* < 0.05 Figure 3A) and females (*p* < 0.01, Figure 3B). *CIDEC* expression was negatively correlated with fasting glucose in females only (r = −507, *p* < 0.01). Leptin expression was significantly lower in the IF and IF+HIIT groups compared to the CON group in both males and females (Figure 3C,D *p* < 0.01) and to the HIIT group in males only (*p* < 0.05) (Figure 3C). Leptin expression was positively correlated with body fat mass in males (r = 0.785, *p* < 0.01) and females (r = 0.730, *p* < 0.01), glucose area under the curve (AUC) in males only (r = 0.511, *p* < 0.01), plasma LDL level in males (r = 0.600, *p* < 0.01) and females (r = 0.364, *p* < 0.05), and plasma HDL level in females (r = 0.424, *p* < 0.05). *PPARγ* expression was significantly higher in the IF+HIIT group compared to the CON (*p* < 0.01) and IF groups in males only (*p* < 0.05, Figure 3E). *PPARγ* expression was negatively correlated with fat mass (r = −0.454, *p* < 0.05), glucose AUC (r = −0.394, *p* < 0.05) and plasma LDL (r = −0.396, *p* < 0.05) in males; and plasma HDL (r = −0.424, *p* < 0.05) in females. *TRPV4* expression was significantly lower in the IF (*p* < 0.01) and IF+HIIT groups (*p* < 0.05) compared to the CON group in males and females (Figure 3O,P). Similarly, lower expression was observed for *FOXO1*, but only in females (Figure 3H). TRPV4 expression in females was negatively correlated to HOMA-IR (r = −0.383, *p* < 0.05). *HADH* expression was significantly lower in the IF group compared to the IF+HIIT group for both males and females (*p* < 0.05, Figure 3I,J). *HADH* expression was negatively correlated with fasting glucose in females only (r = −0.484, *p* < 0.01) and HOMA-IR in both males (r = −0.451, *p* < 0.05) and females (r = −0.384, *p* < 0.05). *FABP4* expression was significantly higher in the IF+HIIT group compared to the CON, HIIT and IF groups in males only (*p* < 0.01, Figure 3K). *FABP4* expression was negatively correlated with body weight (r = −0.596, *p* < 0.01), fat mass (r = −0.573, *p* < 0.01), fasting glucose (r = −0.417, *p* < 0.05), glucose AUC (r = −0.416, *p* < 0.05), HOMA-IR (r = −387, *p* < 0.05) and plasma LDL (r = −0.490, *p* < 0.01) in males. SIRT1 expression was significantly lower in the IF group but only compared to IF+HIIT in males (*p* < 0.05). However, in females, SIRT1 expression in both the IF and IF+HIIT groups was significantly lower compared to the CON (*p* < 0.01) and HIIT (*p* < 0.05) groups. SIRT1 expression was negatively correlated to fasting glucose (r = −0.397, *p* < 0.05) and HOMA-IR (r = −0.375, *p* < 0.05). *HIF1α* expression was significantly lower in the HIIT, IF and IF+HIIT groups compared to the CON group in males only ((*p* < 0.01) Figure 3Q). *HIF1α* expression was positively correlated with fat mass in both males (r = 0.685, *p* < 0.01) and females (r = 0.348, *p* < 0.05), as well as body weight (r = 0.658, *p* < 0.01), glucose AUC (r = 0.484, *p* < 0.05) and plasma LDL (r = 0.679, *p* < 0.01), but only in males.

### 3.6. The Effects of the HF/S Diet on White Adipose Tissue microRNA Expression

**Figure 4:**
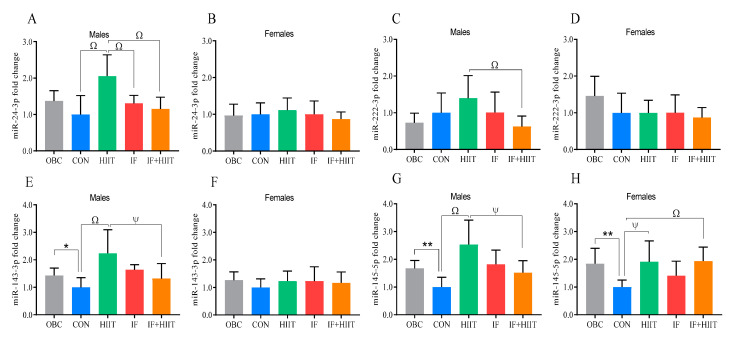
White adipose tissue miRNA expression of the OBC, IF, HIIT and IF+HIIT groups relative to CON. Relative miRNA expression of miR-24 in (**A**) males and (**B**) females, miR-222 in (**C**) males and (**D**) females, miR-143 in (**E**) male and (**F**) females and miR-145 in (**G**) males and (**H**) females. MiRNA expression is normalized to *RNU6* gene expression. Bars represent the mean, and error bars represent the standard deviation. * and ** represent a significant difference between the OBC and the CON group at *p* < 0.05 and *p* < 0.01, respectively. Ψ and Ω represent a significant difference between the CON and IF, HIIT and IF+HIIT groups at *p* < 0.05 and *p* < 0.01, respectively.

Following an additional 12 weeks of the HF/S diet, miR-143 was significantly lower in the CON group compared to the OBC group in males only (*p* < 0.05), Figure 4E), whereas both males and females showed significantly lower levels of miR-145 in the CON group compared to the OBC group (*p* < 0.01, Figure 4G,H).

### 3.7. The Effects of IF and/or HIIT on White Adipose Tissue microRNA Expression

MicroRNA-24, miR-143 and miR-145 expression was significantly higher in the HIIT group compared to the CON group (*p* < 0.01) in male mice only at the end of the 12 week intervention (Figure 4A,E,G). Male mice in the HIIT group also showed higher expression of miR-24 (*p* < 0.01), miR-222 (*p* < 0.01, Figure 4C), miR-143 (*p* < 0.05) and miR-145 (*p* < 0.05) in comparison to the IF+HIIT groups. MiR-24 was also significantly higher in the HIIT group compared to the IF group (*p*< 0.01, Figure 4A). In female mice, significantly higher expression of miR-145 in the HIIT (*p* < 0.05) and IF+HIIT (*p* < 0.01) groups compared to the CON group (Figure 4H) was observed at the end of the 12 week intervention. No significant correlations between miRNAs and their target genes were found (Table S6).

### 3.8. The Effect of the HF/S Diet on EDL Muscle mRNA Expression

**Figure 5:**
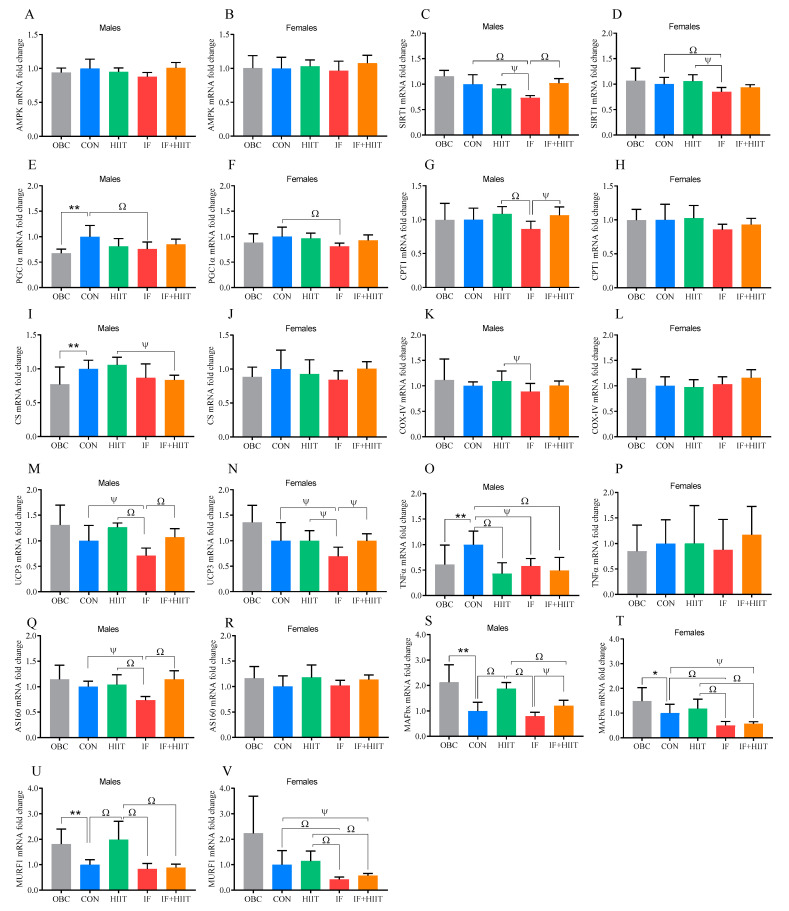
Skeletal muscle (EDL) gene expression of the OBC, IF, HIIT and IF+HIIT groups relative to CON. Relative gene expression of *AMPK* in (A) males and (**B**) females, *SIRT1* in (**C**) males and (**D**) females, *PGC1α* in (**E**) male and (**F**) females, *CPT1* in (**G**) males and (**H**) females, *CS* in (**I**) males and (**J**) females, *COX* IV in (**K**) males and (**L**) females, *UCP3* in (**M**) males and (**N**) females, *TNFα* in (**O**) males and (**P**) females *AS160* in (**Q**) males and (**R**) females, *MAFbx* in (**S**) males and (**T**) females and *MURF1* in (**U**) males and (**V**) females. Gene expression is normalized to *PPIA* gene expression. Bars represent the mean, and error bars represent the standard deviation. * and ** represent a significant difference between the OBC and the CON group at *p* < 0.05 and *p* < 0.01, respectively. Ψ and Ω represent a significant difference between the CON and IF, HIIT and IF+HIIT groups at *p* < 0.05 and *p* < 0.01, respectively.

Following an additional 12 weeks of the HF/S diet, *PGC1a*, *CS* and *TNFα* expression was significantly higher in the CON group compared to the OBC group (*p* < 0.01, Figure 5E,I,O), whereas *MAFbx* and *MURF1* expression was significantly lower in the CON group compared to the OBC group in males (*p* < 0.01, Figure 5S,U). In females, *MAFbx* was also significantly lower in the CON group compared to the OBC group (*p* < 0.05, Figure 5T).

### 3.9. The Effects of IF and/or HIIT on EDL Muscle mRNA Expression

*SIRT1* expression was significantly lower in the IF group compared to the HIIT and CON group in males (*p* < 0.05, *p* < 0.01, respectively, Figure 5C) and females (*p* < 0.05, *p* < 0.01, respectively, Figure 5D), and the IF+HIIT group in males only (*p* < 0.01) at the end of the 12 week intervention. *SIRT1* expression was negatively correlated with fasting glucose in both males (r = −0.384, *p* < 0.05) and females (r = −0.350, *p* < 0.05). *PGC1α* expression was also significantly lower in the IF group compared to the CON group in both males and females (*p* < 0.05, Figure 5E,F). In males, *CPT1* expression was significantly lower in the IF group compared to the HIIT (*p* < 0.01) and IF+HIIT (*p* < 0.05) groups (Figure 5G). Similarly, *COX*-*IV* expression was significantly lower in the IF group, but only compared to the HIIT group (*p* < 0.05, Figure 5K). *CS* expression was significantly lower in the IF+HIIT group compared to the HIIT group in males only (*p* < 0.05, Figure 5I). In females, CS expression was negatively correlated to HOMA-IR (r = −0.343, *p* < 0.05). In males, both *UCP3* and *AS160* expression was significantly lower in the IF group compared to the HIIT (*p* < 0.01), IF+HIIT (*p* < 0.01) and CON (*p* < 0.05) groups (Figure 5M,Q). *AS160* levels were negatively correlated with fasting glucose in both males (r = −0.425, *p* < 0.05) and females (r = −0.373, *p* < 0.05) and glucose AUC in both males (r = −0.508, *p* < 0.01) and females (r = −0.366, *p* < 0.05). In females, *UCP3* expression in the IF group was also significantly lower compared to all other intervention groups (*p* < 0.05, Figure 5N). *TNFα* expression was significantly higher in the CON group compared to the HIIT (*p* < 0.01), IF (*p* < 0.05) and IF+HIIT groups (*p* < 0.01, Figure 5O) in males only. *MAFbx* and *MuRF1* expression was significantly higher in the HIIT group compared to all other intervention groups (*p* < 0.01, Figure 5S,U) in males. IF+HIIT also displayed significantly higher *MAFbx* expression compared to the IF group in males (*p* < 0.05). In females, both *MAFbx* and *MuRF1* expression was lower in the IF group compared to the CON and HIIT groups (*p* < 0.01, Figure 5T,V). Likewise, *MAFbx* and *MuRF1* expression was lower in the IF+HIIT group compared to the CON (*p* < 0.05) and HIIT groups (*p* < 0.01).

### 3.10. The Effects of the HF/S Diet on EDL Muscle microRNA Expression

**Figure 6:**
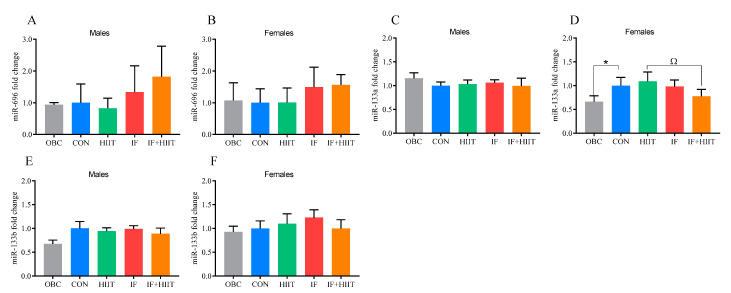
Skeletal muscle (EDL) miRNA expression of the OBC, IF, HIIT and IF+HIIT groups relative to CON. Relative miRNA expression of miR-696 in (**A**) males and (**B**) females, miR-133a in (**C**) males and (**D**) females and miR-133b in (**E**) male and (**F**) females. MiRNA expression is normalized to *RNU6* gene expression. Bars represent the mean, and error bars represent the standard deviation. * represents a significant difference between the OBC and the CON group at *p* < 0.05. Ω represents a significant difference between the CON and IF, HIIT and IF+HIIT groups at *p* < 0.01.

Following an additional 12 weeks of the HF/S diet, miR-133a was significantly higher in the CON group compared to the OBC group in females only (*p* < 0.05, Figure 6D).

### 3.11. The Effects of IF and/or HIIT on EDL Muscle microRNA Expression

MiR-133a was significantly lower in the IF+HIIT group compared to the HIIT (*p* < 0.01) group in females (Figure 6D), with no significant changes observed in males. No other significant changes were identified (p >0.05, Figure 6A–C,E–F). Further, miR-133a expresion was significantly negatively correlated to TNFα gene expression (r = −0.404, *p* < 0.05) (Table S7).

## 4. Discussion

The objective of this investigation was twofold: (1) to assess changes in mRNA expression within the hypothalamus, and mRNA/microRNA expression within adipose and skeletal muscle tissue in response to IF, HIIT and the combination of both; and (2) to identify genes and their regulator that could be involved in the prevention of weight gain and improved lipid profiles observed in our previously published paper [10]. In the hypothalamus of female mice, genes involved in energy homeostasis and obesity development, as well as inflammation, were lower following IF+HIIT and correlated to body weight and fat mass. Within the adipose tissue, both males and females demonstrated lower expression of genes that were positively correlated with fat mass following IF and IF+HIIT. Males demonstrated higher expression of genes involved in fatty acid oxidation following IF+HIIT and, to a lesser degree, IF, which were correlated with lower fat mass, lipid and glucose levels. Conversely, females demonstrated lower expression of genes involved in adipocyte differentiation and storage following IF and/or IF+HIIT which was correlated to higher glucose levels. However, the effects of diet and/or exercise were not observed in all tissues, with limited impact, statistically speaking, observed in the skeletal muscle. Prolonged IF may have a negative impact on glucose tolerance by downregulating genes involved in metabolic signalling and mitochondrial function in the skeletal muscle when concurrently consuming a HF/S diet in both males and females. The current findings demonstrate both sexual-dimorphic and tissue-specific adaptations within diet-induced obese mice following 12 weeks of IF and/or HIIT and while concurrently consuming a HF/S diet. Future work is needed to confirm whether the observed gene changes are driving the body compositional and metabolic improvements or are just a reflection of the current weight (i.e., lower body and fat mass) and/or inflammation status.

In order to determine the directional change in mRNA and microRNA expression due to continued HF/S feeding over the 12 week intervention period, we compared expression levels at 24 weeks (CON) to the 12 week “obese baseline” group (OBC) within all three tissues. Continued HF/S feeding appeared to have little impact in the hypothalamus, with males demonstrating significantly lower expression of genes related to food intake and energy expenditure (*AgRP* and *BDNF*) compared to the OBC group, whereas females demonstrated significantly higher expression of genes related to obesity status and inflammation (*NPY* and *ILβ*) compared to the OBC group, which supports previous observations [12,13]. In the adipose tissue, there were clear sexual-dimorphic effects observed between groups. In males, seven out of the nine genes examined (namely, *CIDEC, PPARγ, HADH, FABP4, SIRT1, FOXO1* and *TRPV4*) showed significantly lower expression in the CON group compared to the OBC group. Lower expression of these genes could relate to reduced adipose non-esterified fatty acid efflux, possibly due to decreased lipolysis/oxidation, lipid accumulation and/or insulin resistance as a result of continued HF/S feeding [14,15,16]. In female mice, leptin and *FOXO1* were significantly higher in the CON group compared to the OBC group. The higher leptin mRNA in females only could be a reflection of differences in fat mass. However, fat mass was similar between groups and continued to increase in both males and females compared to the OBC group (Figure 1C,D). On the other hand, the non-significant change in males could be a reflection of a decrease in leptin sensitivity (and possible downregulation) previously observed in males with long-term high-fat feeding [17]. *FOXO1* isoform is known to play a key role in adipogenesis. However, its physiological role in differentiated adipose tissue remains unclear. In the skeletal muscle, *PGC1α*, *CS* and *TNFα* expression was significantly higher in the CON group compared to the OBC group in male mice only. Additionally, lower expression of markers of atrophy were observed in both males and females, though no differences in lean muscle mass was evident between groups [10]. Changes in *PGC1α* and *CS* in response to high-fat feeding and obesity have been mixed, with some studies demonstrating an increase [18] and others showing a decrease [19]. Differences could be due to strain of animal, diet composition, and importantly intervention duration, with acute high-fat diet shown to downregulate genes involved in mitochondrial biogenesis as well as reduce oxygen capacity, whereas longer term feeding may increase [20,21]. Despite higher expression levels of muscle *PGC1α* and *CS*, high-fat diet-induced insulin resistance can still occur, as it is independent of changes in skeletal muscle mitochondrial (fat) oxidative capacity or muscle mitochondria function [22]. Evidence suggests that male C57BL/6J mice are more susceptible to diet-induced obesity and are likely to present the negative effects of a HF/S diet earlier compared to female mice which are typically impacted to a lesser extent and in a much slower manner [23]. This could be due to females having a higher capacity for storing fat in adipose tissue and oxidizing fatty acids in muscle [24]. Indeed, OBC male mice displayed significantly higher glucose intolerance (glucose AUC), and monophasic glucose curves following glucose tolerance test compared to females after 12 weeks of the HF/S diet. Sex differences were still present and generally enhanced after 24 weeks of the HF/S diet. Male mice demonstrated significantly higher fasting glucose compared to OBC, whereas female mice demonstrated significantly higher insulin levels and markers of insulin resistance compared to OBC. Whether greater (and earlier) susceptibility to a HF/S diet in male mice compared to female mice explains the contrasting changes in some genes expressed, especially within the adipose tissue, requires further investigation. Since the current investigation was unable to compare between 0 week and 12 week, the authors cannot comment on changes within the first 12 weeks. Notwithstanding, several miRNAs, including miR-143, exhibit inverse patterns of regulation, with upregulation in pre-adipocytes (cell culture model) and downregulation in mature adipocytes (animal model) [25]. The contrasting changes in miRNAs, specifically the downregulation in later stages, could be due to the chronic local inflammation environment and enhanced *TNFα* levels often observed in adipose tissue of an obese phenotype. Although we did not measure *TNFα* expression in adipose tissue in the current study, we did measure levels within skeletal muscle and hypothalamus, with males demonstrating significantly higher levels in the CON group compared to the OBC group in skeletal muscle, but not the hypothalamus. 

Hypothalamic *POMC* and *NPY*/*AgRP* neurons are critical nodes of a circuit that sense key metabolic cues as well as regulate metabolism. Elevated hypothalamic *NPY* and decreased *POMC* are thought to promote the development and maintenance of obesity [13]. The effects of calorie restriction and fasting appear to be inconsistent with 12 weeks of moderate continuous restriction (18%) in mice, demonstrating no significant effect on hypothalamic *NPY* or *AgRP* expression, but causing a significant increase in hypothalamic *POMC* expression [26]. Conversely, 3 weeks of a more severe restriction in the form of IF increased expression of *AGRP* and *NPY* in the hypothalamus of Sprague–Dawley rats [27]. While the cellular properties of these neurons have been investigated in the context of obesity and diet, much less is known about the effects of exercise training. Recently, repeated bouts of HIIT training (3 × 20 min) demonstrated, in a time-dependent fashion, an inhibitory effect on *NPY*-expressing neurons while eliciting an overall excitatory effect in adjacent *POMC*-expressing neurons [28]. Conversely, others have shown no effect on *NPY* and *POMC* mRNAs expression levels and food intake after one month of high-intensity exercise [29]. In the current investigation, only the combination of IF+HIIT significantly influenced the expression of hypothalamic *NPY* and *POMC*. *NPY* expression was significantly reduced following IF+HIIT compared to CON in females, whereas *POMC* was significantly increased following IF+HIIT in both males and females, but only significantly when compared to HIIT. *NPY* expression was positively correlated with body weight and fat mass at the end of the intervention period. Decreased expression of *NPY* and to a lesser degree, increased expression of *POMC*, could potentially contribute to the attenuation of weight gain when undertaking IF and HIIT despite concurrent consumption a HF/S diet. However, further work is needed to explore such a concept.

Adipose tissue mass is determined by the storage and removal of triglycerides in adipocytes. Adipocyte lipid turnover, however, is strongly related to conditions with disturbed lipid metabolism. In obesity, the triglyceride removal rate (lipolysis followed by oxidation) is decreased and the amount of triglycerides stored each year is increased. It was evident from the CON group, compared to 12 week OBC, that markers of adipocyte differentiation (*PPARγ*), β-oxidation (*HADH*) and lipid removal (*FABP4*) were lower, especially in males. Reduction of both triglyceride storage and removal decreases lipid shunting through adipose tissue and can promote dyslipidaemia [30], which supports evidence that males are more susceptible to diet-induced obesity compared to females. Notwithstanding the sexual-dimorphic observations in the CON group, similar responses were observed between sexes for leptin and *TRPV4* in response to diet and/or exercise intervention. Lower expression of leptin and *TRPV4* was observed following IF and IF+HIIT compared to CON. Lower levels of leptin are usually a reflection of lower adipose tissue mass [31]. Indeed, leptin expression was positively correlated with body fat mass in males and females. Leptin expression is negatively regulated by *PPARγ* [32] and a significant negative correlation (r = −0.414, *p* = 0.05) was found between *PPARγ* and leptin gene expression in males in the present investigation. Higher levels of *PPARγ*, especially in the IF+HIIT male group, could be due to higher levels of FABP4, which carries fatty acids from the cytoplasm to the nucleus and can act as a *PPARγ* ligand [33]. Indeed, *FABP4* expression was increased in IF+HIIT males compared to all other groups. Previous studies have shown enhanced *FABP4* expression after moderate exercise in both the epididymal and retroperitoneal adipose tissues, suggesting an improvement in the morphometric parameters of the adipose tissue [34]. Increased *FABP4* and *PPARγ* expression in the IF+HIIT group could be contributing to the superior anthropometric benefits observed in our previous study [10], with higher expression levels correlated with lower fat mass. Another important contributor to reduced fat mass in males could be *HIF1α*, with the present study demonstrating lower expression levels in all intervention groups compared to CON, albeit only significantly in males after IF and IF+HIIT. *HIF1α*, a marker of hypoxia [35], is also known to inhibit fatty acid oxidation and energetic uncoupling via transcriptional repression of sirtuin 2 (Sirt2). Adipose tissue hypoxia-induced HIF1α activation suppresses the Sirt2-NAD+ metabolic regulatory system, leading to reduced oxidative lipid catabolism and mitochondrial biogenesis mediated in part through the inhibition of *PGC1α* activity [36]. Although the current investigation did not measure PGC1α or HIF1α protein levels in adipose tissue, we did measure expression of *HADH*, a rate-limiting enzyme of mitochondrial β-oxidation [37]. Expression of *HADH* was significantly higher following IF+HIIT in both males and females, though it was only statistically significant compared to the IF group. 

PPARγ also cooperates with FOXO1 in pre-adipocytes to induce a post-mitotic growth arrest for subsequent differentiation [38]. FOXO1 modulates energy homeostasis in WAT and brown adipose tissue (BAT) through regulation of adipocyte size and adipose tissue-specific gene expression in response to excessive calorie intake. In the present study, only female mice demonstrated lower expression of *FOXO1* following IF and IF+HIIT compared to the CON. Reduced *FOXO1* expression may suggest a decrease in transition from clonal expansion (i.e., cell cycle) to terminal differentiation in pre-adipocytes [38], and while this may occur with advancing obesity and dyslipidaemia, as seen with our CON males compared to females, it may also be further reduced as a result of fasting with or without exercise. *TRPV4* expression was another gene modified by both IF and IF+HIIT in both males and females. The role of *TRPV4* in obesity and obesity development is sometimes conflicting, with knockout of *TRPV4* shown to increase weight gain and promote obesity during high-fat treatment in mice [39], whereas others have shown that administration of GSK205, an inhibitor of *TRPV4*, upregulates the expression level of thermogenic genes such as *PGC1α* and *UCP1*, which further promotes the browning process in 3T3-F442A adipocytes [40]. Indeed, our CON group demonstrated significantly lower expression of *TRPV4* compared to the OBC group, and similarly, our IF and IF+HIIT groups demonstrated significantly lower expression compared to CON. The contradictory role of *TRPV4* in adipogenesis and obesity requires further research, especially regarding the effects of lifestyle intervention on its expression.

In skeletal muscle, *SIRT1*, *PGC1α* and *UCP3* was significantly lower in both males and females following IF compared to CON and, in some cases, compared to HIIT. Reduced levels or compromised activity of *PGC1α* can be associated with the development of insulin resistance and Type 2 diabetes [41]. Acute high-fat feeding for 3 days has been shown to downregulate *PGC1α* expression in skeletal muscle [18], while 22 days of alternate day fasting had no significant effect on expression levels [42]. Conversely, exercise has been shown to enhance expression of *PGC1α* [43]. In the present study, 12 weeks of HIIT was unable to significantly affect *PGC1α* expression levels, which may be due to the lower exercise volume used compared to others [43]. However, it is possible that the HF/S diet consumed while training is negating any beneficial molecular changes from exercise as reported in previous studies [44]. This could also be the reason why IF was unable to have a positive effect on markers of fatty acid metabolism with persistent overload of fatty acids to skeletal muscle as a result of consuming a HF/S diet creating a metabolic gridlock and 2 days of fasting unable to sufficiently improve. IF did result in a worsening of glucose tolerance in both males and females [10] and SIRT1 and AS160 expression was significantly correlated to fasting glucose and/or glucose tolerance and thus could play a contributing role. Lower expression of atrophy markers *MURF1* and *MAFbx* was observed following IF with or without HIIT in females, which may indicate a protective effect on lean muscle mass, as previously reported in other studies [45,46]. Conversely, HIIT induced higher expression of these markers, albeit only significantly compared to CON in the male mice. This could be a reflection of greater remodelling rather than muscle atrophy per se. Indeed, higher expression of *MURF1* and *MAFbx* after 8 weeks of exercise training has previously been reported and associated with higher skeletal muscle turnover rather than an indicator of greater atrophy [47]. Any benefits within skeletal muscle could be driven by lower inflammation levels, with *TNFα* expression significantly lower in all intervention groups (*p* < 0.01). Though not observed in female mice, the observations in males is in line with previous human [48] and animal studies [49].

## 5. Limitations

There are several limitations that exist in this study. Firstly, given the invasive nature of sample collection, the current investigation was unable to compare within-subject changes, as only post-intervention samples could be excised and analysed. However, to gain an understanding in the direction of mRNA and miRNA expression, we compared samples obtained from one group at 12 weeks to the CON group at 24 weeks. We understand that this is a limitation, given that samples were not obtained from the same animal. Secondly, our investigation did not have a ‘true’ baseline sample (prior to HF/S feeding) and no samples were obtained prior to 12 weeks of HF/S feeding. Furthermore, we did not have a standard chow diet control. Thus, we cannot comment on the acute molecular changes as a result of diet only, as the confounding effects of obesity status are most likely present at 12 weeks. Given that mRNA and miRNA expression can be transient and sometimes inverse when compared acutely and chronically, we were unable to investigate this, especially any sexual-dimorphic differences, as mentioned in the manuscript. Thirdly, our investigation only focused on expression of mRNA and miRNA and thus it unclear whether similar changes are occurring at the protein and/or function level. It is evident that changes in some mRNAs may not reflect changes in the protein and/or function level, but also, inverse changes may occur between mRNA and protein/function. Finally, miRNAs targeted in the present study were selected on the basis of their correlation having been already reported (literature or in silico), and most of them did not show any significant correlation to the targeted gene. This does not simply imply their non-involvement in obesity but needs more thorough investigation to establish their role.

## 6. Conclusions

**Figure 7:**
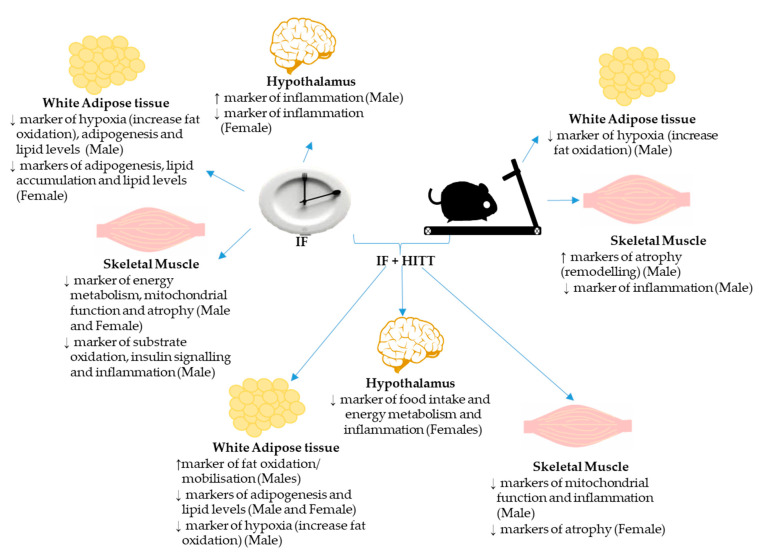
Summary of molecular changes following IF, HIIT and IF+HIIT compared to HF/S control within hypothalamus, white adipose tissue and skeletal muscle.

The major findings of the study are summarised in Figure 7. The combination of IF and HIIT appeared to have the greatest effect on gene expression, especially within adipose tissue and in males, compared to either intervention alone. There were clear sexual-dimorphic effects, especially within adipose tissue, with males demonstrating higher markers of fatty acid oxidation, rather than a reduction in adipogenesis/storage, as observed in females. Correlations between genes *PPARγ*, *FABP4* and/or *HIF1*α expressed in adipose tissue and lower levels of fat mass, plasma LDL levels and glucose observed in our previous paper [10] suggest potential key molecular pathways that could be underpinning the greater improvements in IF+HIIT compared to other groups. This could be a reflection of the greater energy deficit created by the two interventions when combined. However, given fat depots between males and females are distinct anatomical entities and the possible existence of sex-dependent recruitment and modulation of peri-ovarian and epididymal fat depots, caution should be taken when interpreting and comparing diet- and exercise-induced changes in WAT mRNA expression between males and females. Interestingly, the HF/S diet appeared to negatively impact skeletal muscle, especially in those undertaking IF, which may be a result of increased fat delivery/supply to muscle from the diet but also fasting-induced ketosis during metabolic slowdown. This investigation is unlike other studies, we examined the impact of a lifestyle intervention on diet-induced obese mice but with continued consumption of a very-high-fat and sugar diet. Therefore, based on the body composition, metabolic and gene expression data, the beneficial effects of IF and HIIT are limited. Thus, future research should determine whether the widely touted benefits of IF and HIIT are still evident while concurrently consuming a high-fat and sugar diet (i.e., a typical Western diet), or whether diet also needs to be changed when undergoing such interventions. Notwithstanding, it is clear that adaptions are sexual-dimorphic and tissue-specific, with inconsistent changes observed at the end of the intervention. Understanding changes at a molecular level can help provide mechanisms for the body composition and metabolic adaptations that typically occur with such lifestyle interventions and, importantly, may help identify key physiopathological networks in order to design suitable therapeutic targets for the treatment of obesity and associated disorders. 

## Supplementary Materials

The following are available online at https://www.mdpi.com/2072-6643/12/6/1764/s1, Figure S1: Change in body weight over the 10-weeks intervention period in (A) male and (B) females. Data is presented as mean of each group and error bars represent standard deviation, Table S1: List of targeted genes in with full official names, NCBI reference sequence numbers and Qiagen catalogue number, Table S2: List of miRNAs analysed in adipose tissue, miRbase accession numbers and Qiagen miScript Primer Assay Catalog Numbers, Table S3: Pearson correlation between hypothalamus gene markers with body composition and glucose parameters, Table S4: Pearson correlation between adipose tissue gene markers with body composition, glucose parameters and plasma lipids, Table S5: Pearson correlation between muscle gene markers with body composition and glucose parameters, Table S6. Pearson correlation between mRNA and miRNA expression in adipose tissue, Table S7. Pearson correlation between mRNA and miRNA expression in muscle.

## Abbreviations

ACTB	βActin
AGRP	Agouti-related protein
AMPK	Protein kinase
AMP	Activated catalytic subunit alpha 2
AS160	Akt substrate of 160kDa
B2M	β2 microglobulin
BDNF	Brain-derived neurotrophic factor
BiP	Binding immunoglobulin protein
CIDEC	Cell death-inducing DFFA-like effector c
COX-IV	Cytochrome c oxidase subunit 4 isoform 2
CPT1	Carnitine palmitoyltransferase Ib
CS	Citrate synthase
FABP4	Fatty acid-binding protein 4
FOXO1	Forkhead box O 1
FTO	Fat mass and obesity associated
GAPDH	Glyceraldehyde-3-phosphate dehydrogenase
HADH	Hydroxyacyl-coenzyme A dehydrogenase
HIF1α	Hypoxia-inducible factor 1α
IL1β	Interleukin 1β
LEP	Leptin
MAFbx	F-box protein 32
MuRF1	Muscle RING finger 1
NPY	Neuropeptide Y
PGC1α	Peroxisome proliferator-activated receptor gamma coactivator 1α
POMC	Pro-opiomelanocortinα
PPARγ	Peroxisome proliferator-activated receptor γ
PPIA	Peptidylprolyl isomerase A
SIRT1	Sirtuin 1
TNFα	Tumor necrosis factor α
TRPV4	Transient receptor potential cation channel, subfamily V, member 4
UCP1	Uncoupling protein 1
UCP3	Uncoupling protein 3
XBP1us	X box binding protein 1 (unspliced)
XBP1s	X box binding protein 1 (spliced)
