# Supplementary material for: Intermittent Fasting and High-Intensity Exercise Elicit Sexual-Dimorphic and Tissue-Specific Adaptations in Diet-Induced Obese Mice
Source: Nutrients. 2020 Jun 12;12(6):1764. doi: 10.3390/nu12061764 (PMC7353251; doi:10.3390/nu12061764)
Supplement: Supplementary file 1 [file nutrients-12-01764-s001.pdf]

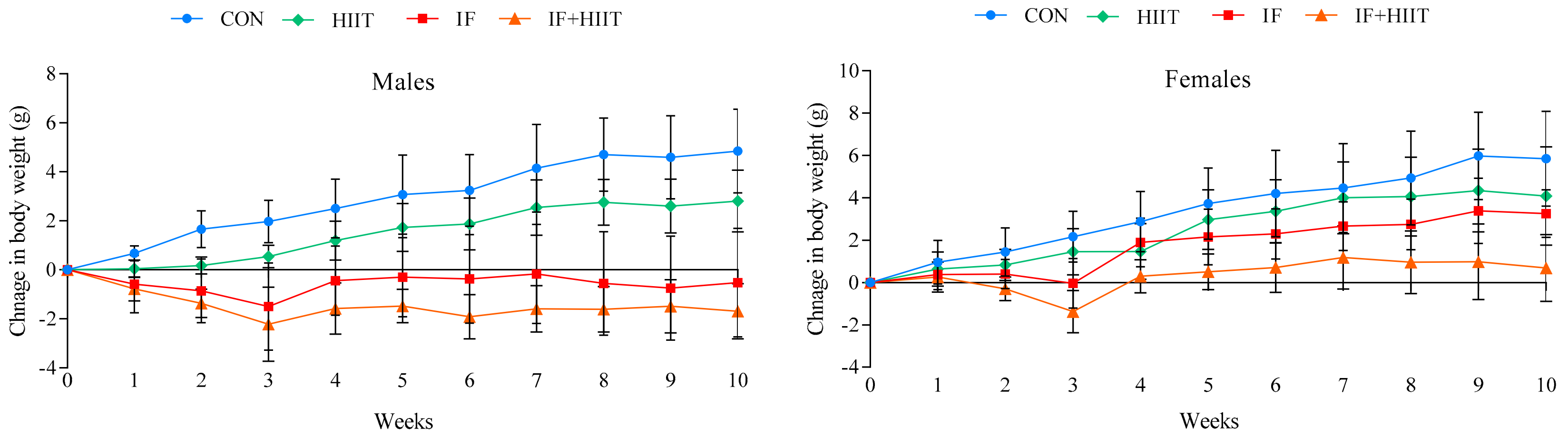

Figure S1. Change in body weight over the 10-weeks intervention period in (A) male and (B) females. Data is presented as mean of each group and error bars represent standard deviation.

Table S1. List of targeted genes in with full official names, NCBI reference sequence numbers and Qiagen catalogue number.

***Gene* symbol**	**Official full name**	**Marker**	**NCBI reference sequence number**	**Qiagen Catalogue number**
ACTB	β-Actin, beta	House keeping	NM_007393	PPM02945
AGRP	Agouti related protein	Orexigenic	NM_007427	PPM29573
AMPK	Protein kinase AMP-activated catalytic subunit α2	Energy sensor	NM_178143	PPM29410F
AS160	Akt substrate of 160 kDa	Insulin signalling	NM_00108127	PPM40317A
B2M	Beta-2 microglobulin	House keeping	NM_009735	PPM03562A
BDNF	Brain derived neurotrophic factor	Neuronal plasticity	NM_007540	PPM03006
BiP	Binding-Immunoglobulin Protein	Endoplasmic reticulum stress	NM_022310	PPM03586
CIDEC	Cell death-inducing DFFA-like effector c	Unilocular lipid droplet formation	NM_178373	PPM25558B
COX-IV	Cytochrome c oxidase subunit 4 isoform 2	Oxidative metabolism	NM_053091	PPM32878E
CPT1	Carnitine palmitoyltransferase Ib	Oxidative metabolism	NM_009948	PPM57688A
CS	Citrate synthase	Oxidative metabolism	NM_026444	PPM29621B
FABP4	Fatty acid binding protein 4, adipocyte	Fat mobilization	NM_024406	PPM04517A
FOXO1	Forkhead box O 1	Adipocyte differentiation and maturation	NM_019739	PPM03381C
FTO	Fat mass and obesity associated	Obesity	NM_011936	PPM39276
GAPD	Glyceraldehyde-3-phosphate dehydrogenase	House keeping	NM_008084	PPM02946
HADH	Hydroxyacyl-Coenzyme A dehydrogenase	β Oxidation	NM_008212	PPM35732B
HIF1α	Hypoxia inducible factor 1, alpha	Hypoxia	NM_010431	PPM03799C
IL1β	Interleukin 1 beta	Inflammation	NM_008361	PPM03109
LEP	Leptin	Obesity	NM_008493	PPM03504B
MAFbx	F-box protein 32	Muscle remodelling/atrophy	NM_026346	PPM38061A
MuRF1	Muscle RING finger 1	Muscle remodelling/atrophy	NM_00103904	PPM61645B
NPY	Neuropeptide Y	Orexigenic	NM_023456	PPM04323
PGC1α	Peroxisome proliferator-activated receptor gamma coactivator 1-alpha	Energy sensing & mitochondrial function	NR_027710	PPM03360I
POMC	Pro-opiomelanocortin-alpha	Anorexigenic	NM_008895	PPM37114
PPARγ	Peroxisome proliferator activated receptor γ	Adipocyte differentiation & maturation	NM_001127330	PPM05108C
PPIA	Peptidylprolyl isomerase A	House keeping	NM_008907	PPM03717B
SIRT1	Sirtuin 1	Energy sensor	NM_001159589	PPM05054A
TNFα	Tumor necrosis factor-alpha	Inflammation	NM_013693	PPM03113G

TRPV4	Transient receptor potential cation channel, subfamily V, member 4	Inflammation	NM_022017	PPM36070A
UCP3	Uncoupling protein 3	Fatty acid metabolism	NM_009464	PPM25342F
XBP1us	X-box binding protein 1 (unspliced)	Endoplasmic reticulum stress	NM_013842	PPM84308A
XBP1s	X-box binding protein 1 (spliced)	Endoplasmic reticulum stress	NM_001271730	PPM84307A

Table S2. List of miRNAs analysed in adipose tissue, miRbase accession numbers and Qiagen miScript Primer Assay Catalog Numbers.

**Mature miRNA ID**	**Gene target**	**miRBase Accession No.**	**Qiagen miScript Primer Assay Catalog No.**
mmu-miR-24-3p	*FABP4*	MIMAT0000219	MS00005922
mmu-miR-222-3p	*HIF1α*	MIMAT0000670	MS00007959
mmu-miR-143-3p	Leptin	MIMAT0000247	MS00001617
mmu-miR-145a-5p	*FOXO1*	MIMAT0000157	MS00001631
mmu-miR-696	*PGC1α*	MIMAT0003483	MS00002870
mmu-miR-133a-3p	*TNFα*	MIMAT0000145	MS00007294
mmu-miR-133b-3p	*MuRF1*	MIMAT0000769	MS00007301

Table S3. Pearson correlation between hypothalamus gene markers with body composition and glucose parameters

			AgRP	NPY	POMC	BDNF	TNFα	IL1B	BiP	FTO	XBPs/XBPus
Body Wt	Males	r	-0.190	0.093	0.091	-0.036	0.318	-0.261	0.321	-0.145	0.248
		p value	*0.343*	*0.639*	*0.651*	*0.850*	*0.093*	*0.180*	*0.083*	*0.446*	*0.178*
	Females	r	-0.188	**0.402**	-0.125	-0.021	0.147	-0.049	-0.096	-0.324	0.140
		p value	*0.266*	*0.014*	*0.504*	*0.900*	*0.386*	*0.773*	*0.585*	*0.058*	*0.395*
Fat Mass	Males	r	-0.090	0.091	0.087	0.014	0.250	-0.313	0.313	0.026	0.237
		p value	*0.657*	*0.646*	*0.667*	*0.942*	*0.192*	*0.104*	*0.093*	*0.890*	*0.199*
	Females	r	-0.198	**0.422**	-0.172	-0.032	0.108	-0.038	-0.092	-0.366	0.146
		p value	*0.241*	*0.009*	*0.356*	*0.849*	*0.524*	*0.821*	*0.599*	*0.031*	*0.375*
Lean mass	Males	r	-0.293	0.013	0.019	-0.139	0.241	0.032	0.116	-0.417	0.099
		p value	*0.138*	*0.950*	*0.925*	*0.464*	*0.208*	*0.870*	*0.541*	*0.022*	*0.596*
	Females	r	-0.057	0.160	0.149	0.081	0.300	-0.061	-0.100	-0.012	0.107
		p value	*0.738*	*0.345*	*0.425*	*0.635*	*0.072*	*0.718*	*0.568*	*0.944*	*0.516*
Fasting glucose	Males	r	-0.188	0.060	-0.053	-0.191	0.237	0.310	0.120	-0.385	0.037
		p value	*0.348*	*0.762*	*0.792*	*0.312*	*0.217*	*0.108*	*0.527*	*0.036*	*0.843*
	Females	r	-0.160	0.037	-0.068	-0.130	0.049	-0.011	0.130	-0.108	-0.008
		p value	*0.344*	*0.828*	*0.718*	*0.444*	*0.775*	*0.946*	*0.455*	*0.537*	*0.961*
Glucose AUC	Males	r	-0.170	0.426	-0.066	-0.227	0.202	0.145	-0.084	-0.317	-0.148
		p value	*0.396*	*0.024*	*0.745*	*0.229*	*0.295*	*0.461*	*0.661*	*0.088*	*0.426*
	Females	r	-0.236	0.093	-0.001	-0.161	-0.030	0.032	-0.053	-0.309	0.344
		p value	*0.159*	*0.586*	*0.997*	*0.341*	*0.859*	*0.850*	*0.760*	*0.071*	*0.032*
HOMA-IR	Males	r	-0.038	-0.060	0.091	-0.121	**0.501**	0.055	0.290	-0.055	0.292
		p value	*0.851*	*0.763*	*0.651*	*0.525*	*0.006*	*0.780*	*0.120*	*0.773*	*0.111*
	Females	r	-0.053	0.113	-0.107	0.032	0.050	-0.060	0.043	-0.075	-0.155
		p value	*0.755*	*0.506*	*0.568*	*0.849*	*0.768*	*0.726*	*0.808*	*0.670*	*0.347*

Table S4. Pearson correlation between adipose tissue gene markers with body composition, glucose parameters and plasma lipids.

			CIDEC	Leptin	PPAR	FOXO	HADH	FABP4	SIRT1	TRPV	HIF1a
Body Wt	Males	r	-0.157	**0.722**	**-0.459**	-0.069	-0.281	**-0.596**	-0.213	0.038	**0.658**
		p value	*0.425*	*0.000*	*0.014*	*0.726*	*0.147*	*0.001*	*0.258*	*0.841*	*0.000*
	Females	r	-0.005	**0.671**	-0.157	0.340	-0.126	-0.112	0.015	0.064	**0.319**
		p value	*0.976*	*0.000*	*0.367*	*0.053*	*0.479*	*0.544*	*0.930*	*0.713*	*0.070*
Fat Mass	Males	r	-0.107	**0.785**	**-0.454**	-0.008	-0.185	**-0.574**	-0.178	0.075	**0.685**
		p value	*0.588*	*0.000*	*0.015*	*0.969*	*0.345*	*0.001*	*0.347*	*0.687*	*0.000*
	Females	r	0.072	**0.730**	-0.129	**0.366**	-0.087	-0.063	0.058	0.106	**0.349**
		p value	*0.692*	*0.000*	*0.462*	*0.036*	*0.625*	*0.733*	*0.735*	*0.541*	*0.047*
Lean mass	Males	r	-0.179	0.107	-0.196	-0.148	-0.324	-0.258	-0.146	-0.051	0.149
		p value	*0.363*	*0.576*	*0.317*	*0.451*	*0.093*	*0.177*	*0.443*	*0.786*	*0.458*
	Females	r	**-0.358**	0.218	-0.226	0.159	-0.290	-0.295	-0.206	-0.149	0.075
		p value	*0.041*	*0.208*	*0.192*	*0.378*	*0.096*	*0.101*	*0.222*	*0.387*	*0.677*
Fasting glucose	Males	r	-0.289	0.098	-0.296	-0.153	-0.373	**-0.417**	-0.343	-0.114	0.137
		p value	*0.136*	*0.605*	*0.126*	*0.437*	*0.051*	*0.024*	*0.064*	*0.542*	*0.495*
	Females	r	**-0.507**	-0.111	-0.328	-0.151	**-0.564**	**-0.412**	**-0.416**	-0.300	0.082
		p value	*0.003*	*0.526*	*0.055*	*0.401*	*0.001*	*0.019*	*0.010*	*0.076*	*0.650*
Glucose AUC	Males	r	-0.030	**0.511**	**-0.394**	0.200	0.049	**-0.416**	-0.187	0.076	**0.484**
		p value	*0.881*	*0.004*	*0.038*	*0.307*	*0.806*	*0.025*	*0.322*	*0.684*	*0.011*
	Females	r	-0.274	0.279	-0.180	0.191	-0.177	-0.229	-0.264	-0.074	0.163
		p value	*0.123*	*0.105*	*0.302*	*0.287*	*0.316*	*0.207*	*0.114*	*0.667*	*0.366*
HOMA-IR	Males	r	-0.306	0.156	-0.160	-0.340	**-0.451**	**-0.387**	-0.076	-0.225	0.087
		p value	*0.114*	*0.411*	*0.417*	*0.077*	*0.016*	*0.038*	*0.689*	*0.225*	*0.666*
	Females	r	-0.283	0.089	-0.130	0.050	**-0.384**	-0.322	**-0.375**	**-0.383**	-0.080
		p value	*0.111*	*0.611*	*0.455*	*0.783*	*0.025*	*0.073*	*0.022*	*0.021*	*0.657*
TAG	Males	r	0.256	-0.121	0.118	0.246	-0.045	-0.127	-0.129	0.270	-0.069
		p value	*0.198*	*0.532*	*0.560*	*0.206*	*0.819*	*0.512*	*0.504*	*0.149*	*0.734*
	Females	r	0.126	0.102	-0.328	0.118	-0.029	0.320	0.229	0.074	0.029
		p value	*0.487*	*0.558*	*0.054*	*0.514*	*0.870*	*0.074*	*0.173*	*0.667*	*0.872*
HDL	Males	r	-0.182	0.067	-0.274	-0.056	-0.166	-0.216	-0.297	-0.112	0.063
		p value	*0.355*	*0.726*	*0.159*	*0.779*	*0.398*	*0.261*	*0.111*	*0.549*	*0.756*
	Females	r	0.132	**0.424**	**-0.424**	0.048	-0.016	-0.081	0.125	0.189	0.186
		p value	*0.464*	*0.011*	*0.011*	*0.791*	*0.928*	*0.658*	*0.460*	*0.271*	*0.300*
LDL	Males	r	0.029	**0.600**	**-0.396**	0.171	-0.077	**-0.490**	-0.089	0.254	**0.679**
		p value	*0.882*	*0.001*	*0.037*	*0.385*	*0.696*	*0.007*	*0.639*	*0.168*	*0.000*
	Females	r	0.169	**0.364**	0.148	0.157	0.201	0.029	0.122	0.187	0.272
		p value	*0.346*	*0.032*	*0.397*	*0.383*	*0.255*	*0.873*	*0.473*	*0.274*	*0.126*

Table S5. Pearson correlation between muscle gene markers with body composition and glucose parameters

			AMPK	SIRT1	PGC1	CPT1	CS	COX4	UCP3	TNFa	As160	MURF	MAFbx
Body Wt	Males	r	0.166	-0.129	**0.491**	-0.127	0.267	0.160	-0.131	**0.502**	-0.280	0.141	-0.154
		p value	*0.400*	*0.505*	*0.006*	*0.510*	*0.161*	*0.408*	*0.514*	*0.007*	*0.149*	*0.485*	*0.434*
	Females	r	0.019	0.044	0.304	0.267	0.031	-0.046	0.187	0.140	-0.199	-0.029	0.023
		p value	*0.913*	*0.806*	*0.076*	*0.105*	*0.861*	*0.796*	*0.283*	*0.444*	*0.267*	*0.867*	*0.898*
Fat Mass	Males	r	0.307	-0.005	**0.492**	-0.028	0.220	0.220	-0.008	**0.498**	-0.206	0.176	-0.029
		p value	*0.112*	*0.978*	*0.006*	*0.887*	*0.252*	*0.251*	*0.968*	*0.007*	*0.294*	*0.379*	*0.883*
	Females	r	0.029	0.111	**0.342**	0.282	0.046	-0.084	0.186	0.110	-0.171	-0.013	0.035
		p value	*0.867*	*0.534*	*0.044*	*0.086*	*0.791*	*0.638*	*0.285*	*0.547*	*0.341*	*0.938*	*0.842*
Lean mass	Males	r	-0.229	-0.294	0.163	-0.266	0.173	-0.099	-0.312	0.174	-0.232	-0.056	-0.326
		p value	*0.240*	*0.122*	*0.390*	*0.163*	*0.369*	*0.609*	*0.113*	*0.377*	*0.235*	*0.782*	*0.091*
	Females	r	-0.029	-0.296	0.017	0.093	-0.084	0.070	0.140	0.199	-0.237	-0.068	-0.024
		p value	*0.863*	*0.089*	*0.925*	*0.578*	*0.633*	*0.696*	*0.423*	*0.275*	*0.184*	*0.692*	*0.890*
Fasting glucose	Males	r	-0.119	**-0.384**	0.158	**-0.403**	-0.028	-0.109	-0.335	0.276	**-0.425**	-0.076	-0.284
		p value	*0.546*	*0.040*	*0.406*	*0.030*	*0.883*	*0.575*	*0.087*	*0.155*	*0.024*	*0.707*	*0.143*
	Females	r	**-0.337**	-0.350	-0.129	-0.234	-0.301	-0.074	-0.222	0.077	**-0.373**	-0.272	-0.254
		p value	*0.042*	*0.042*	*0.459*	*0.157*	*0.080*	*0.676*	*0.200*	*0.677*	*0.033*	*0.108*	*0.141*
Glucose AUC	Males	r	0.035	-0.234	0.164	-0.360	-0.036	-0.158	-0.203	0.249	**-0.508**	0.117	-0.094
		p value	*0.859*	*0.221*	*0.388*	*0.055*	*0.852*	*0.413*	*0.311*	*0.201*	*0.006*	*0.561*	*0.635*
	Females	r	-0.167	-0.143	-0.003	-0.110	-0.169	-0.097	-0.052	0.379	**-0.366**	0.032	0.072
		p value	*0.323*	*0.418*	*0.987*	*0.511*	*0.332*	*0.585*	*0.768*	*0.032*	*0.036*	*0.851*	*0.681*
HOMA-IR	Males	r	-0.079	-0.221	0.189	-0.174	0.049	0.012	-0.247	0.210	-0.246	-0.096	-0.204
		p value	*0.690*	*0.249*	*0.319*	*0.367*	*0.799*	*0.952*	*0.214*	*0.283*	*0.207*	*0.633*	*0.297*
	Females	r	-0.115	-0.284	-0.108	-0.070	-0.343	0.011	0.119	-0.206	-0.192	-0.079	-0.144
		p value	*0.499*	*0.104*	*0.537*	*0.678*	*0.044*	*0.950*	*0.496*	*0.258*	*0.285*	*0.649*	*0.408*

Table S6. Pearson correlation between mRNA and miRNA expression in adipose tissue

		Males		Females	
Gene	miRNA	r	P value	r	P value
FABP4	miR-24-3p	0.099	*0.622*	-0.069	*0.713*
HIF1	miR-222-3p	0.189	*0.365*	0.107	*0.568*
Leptin	miR-143-3p	-0.096	*0.627*	-0.295	*0.096*
FOXO1	miR-145-5p	0.476	*0.016*	-0.131	*0.497*

Table S7. Pearson correlation between mRNA and miRNA expression in muscle

		Males		Females	
Gene	miRNA	r	p value	r	p value
PGC1	miR-696	-0.065	*0.752*	0.488	*0.009*
TNF	miR-133a	-0.190	*0.362*	**-0.404**	*0.041*
MURF1	miR-133b	0.105	*0.627*	-0.270	*0.164*
